# Mild Cognitive Decline Is a Risk Factor for Scam Vulnerability in Older Adults

**DOI:** 10.3389/fpsyt.2021.685451

**Published:** 2021-12-20

**Authors:** Daisuke Ueno, Yasuhiro Daiku, Yoko Eguchi, Minako Iwata, Shoka Amano, Nobutaka Ayani, Kaeko Nakamura, Yuka Kato, Teruyuki Matsuoka, Jin Narumoto

**Affiliations:** ^1^Department of Psychiatry, Graduate School of Medical School, Kyoto Prefectural University of Medicine, Kyoto, Japan; ^2^Graduate School of Human Sciences, Osaka University, Osaka, Japan; ^3^Department of Neuropsychiatry, Keio University School of Medicine, Tokyo, Japan; ^4^Senior Consumer Mimamori Club, Sagamihara, Japan; ^5^Department of Psychiatry, Maizuru Medical Center, National Hospital Organization, Kyoto, Japan; ^6^Kyoto Prefectural Mental Health and Welfare Center, Kyoto, Japan

**Keywords:** fraud vulnerability, scams vulnerability, mild cognitive decline, older adult, Japanese version of the Alzheimer's diseases assessment scale-cognitive subscale (ADAS-Jcog)

## Abstract

Research on elderly financial exploitation has mostly focused on financial abuse that occurs in families and other types of trusted relationships. As such, little is known about financial frauds and scams perpetrated by strangers. Financial fraud and scam prevention activities for older adults must be promoted, for which the correlation between the psychological, social, and cognitive characteristics of their vulnerability needs to be determined. The present study aimed to determine whether cognitive decline is a risk factor for scam vulnerability in older adults. Thus, we created a scam vulnerability scale for older adults with cognitive decline and analyzed the data to reveal the correlation between them, including inhibition and executive function. We conducted an interview survey with 50 older adults with cognitive decline (average age: 79.42 years, SD: 5.44) and 51 older adults without cognitive decline (average age: 76.12 years, SD: 5.82). The interview survey included the scam vulnerability scale, psychosocial questionnaires, and neuropsychological tests. The scale included six items with a four-point Likert scale based on a previous study. Hierarchical multiple regression analysis revealed that lower scores on the Japanese version of the Alzheimer's Disease Assessment Scale–Cognitive Subscale (ADAS-Jcog; indicating higher general cognitive function) correlated with higher scam vulnerability in the cognitive decline group (*β* = −0.46, *p* < 0.001). In addition, men were found to be more vulnerable in both groups (cognitive decline group: *β* = −0.29, *p* = 0.015, cognitive non-decline group; *β* = −0.32, *p* = 0.018). Inhibition and executive function were found not to correlate significantly with scam vulnerability. These results suggest that mild cognitive decline correlates with higher scam vulnerability, whereas moderate to severe cognitive decline correlates with lower vulnerability, possibly because it makes understanding the scam attempt itself difficult. Older adults with mild cognitive decline and their families, particularly those visiting elderly care or outpatient facilities, should be notified of the scam vulnerability of older clients using the ADAS-Jcog score as an index to help them avoid victimization.

## Introduction

In Japan, the number of reported scam cases was 16,851 in 2019, amounting to total damage of 31.58 billion JPY (~305.7 million USD) (1). In 83.7% of these cases, the victims were older adults, and there is a more prevalent rate of consumer fraud among older adults compared with the United States (2). As risk factors of financial fraud and scam victimization, Fenge and Lee (3) summarize psychological characteristics (e.g., emotion), social characteristics (e.g., social isolation or loneliness), and cognitive characteristics (e.g., cognitive impairment) from their small exploratory quantitative study in older adults.

With the spread of the Internet, some fraudsters described as “enterprising” or “vocational predators” set up a diversity of fraud and scam schemes (4). Button et al. (4) reported fraudster techniques committed against online fraud victims, including grooming, authority and legitimacy, visceral appeals, embarrassing the victim, or pressure and coercion. Moreover, financial exploitation is associated with not only an economic loss but also increased risks of mortality, hospitalization, poor physical and mental health, and diminished quality of life (5). Fraud and scam victimization is associated with broken relationships within families, thinking about or attempted suicide, and fear of violence or legal action (6). Thus, financial fraud and scam prevention activities must be promoted, and this requires research on the correlation between the psychological, social, and cognitive characteristics and their vulnerability to frauds and scams.

Cognitive decline has been identified as a risk factor for vulnerability to frauds and scams (7, 8). Han et al. (7) measured episodic memory, semantic memory, working memory, perceptual processing speed, and visuospatial ability as general cognitive functions. They (8) reported that older adults with mild cognitive impairment (MCI) have a higher age, lower cognitive function, and higher telephone scam vulnerability. Moreover, older age, years of education, and diagnosis of MCI enhance telephone scam vulnerability. Thus, older adults with MCI have higher scam vulnerability compared with control older adults. In another study, older adults who were victims of financial exploitation have shown slower processing speed and poorer executive functioning through the completion of the Trail Making Test compared with their age-matched control group (9). However, the correlation between scam vulnerability and frontal lobe function, such as inhibition and executive function, remains unclear. Thus far, cyber-fraud victims tend to have a higher age and higher score on impulsivity, such as urgency and sensation seeking (10). As such, control over inhibition and executive function seems to be important for fraud and scam vulnerability.

Both cognitive function and psychosocial characteristics are identified as risk factors that increase fraud and scam victimization or vulnerability. Lichtenberg et al. (11, 12) reported older adults involved in fraud victimization to show a younger old age, more education years, more severe depressive symptoms, and lower income satisfaction than older adults who had not been victimized. Alves and Wilson (13) reported that older adults with experience in telemarketing frauds tend to be divorced or separated, with their marital status correlated with loneliness. Van Wyk and Mason (14) reported that individuals with higher socialization (e.g., participating in community functions) and higher risk-taking tend to be victimized. However, they examined only psychosocial characteristics and did not distinguish or include older adults with cognitive decline.

Moreover, research has examined the correlation between fraud and scam victimization or vulnerability, cognitive function, and psychosocial characteristics. Judges et al. (8) reported that older adults with fraud victimization experience show not only cognitive decline but also personality traits of lower honesty–humility and conscientiousness compared with older adults without victimization experience. Gamble et al. (15) found that older adults with fraud victimization experience demonstrate cognitive decline and overconfidence in their financial knowledge compared with older adults without victimization experience. James et al. (16) confirmed that cognitive decline, poorer well-being, and lower levels of financial and health literacy correlate with higher scam vulnerability, after adjusting for cognitive function in older adults. However, these previous studies did not distinguish or include older adults with an MCI diagnosis. Moreover, Han et al. (7) examined cognitive function but not psychosocial characteristics in older adults with or without MCI.

It is important to measure fraud and scam vulnerability for promoting self-help or interventions to prevent victimization, especially in older adults with cognitive decline. Few scales have been designed for measuring this vulnerability. James et al. (16) developed a telemarketing fraud vulnerability scale for older adults; however, this scale specializes in measuring only telemarketing fraud vulnerability. At present, no scale has been developed to measure fraud and scam vulnerability in older adults with cognitive decline.

The present study aimed to determine whether cognitive decline is a risk factor for scam vulnerability in older adults. Thus, we developed a scam vulnerability scale for older adults with cognitive decline and analyzed the data to reveal the correlation between scam vulnerability and cognitive decline, including inhibition and executive function.

## Methods

### Participants

In this study, we enrolled 50 older adults (mean age = 79.42 years, standard deviation [SD] = 5.44, range = 68–90; 35 women) in the cognitive decline group (CD) and 51 older adults (mean age = 76.12 years, SD = 5.82, range = 62–85; 27 women) in the cognitive non-decline group (CND). The sample size was calculated using G^*^ Power 3.1 (17) for linear multiple regression from the effect size (f^2^) as 0.25, the power (1–β error probability) as 0.8, the significance level (α error probability) as 0.05, and the number of predictors as 20. The calculated sample size was 101 participants.

The participants were outpatients of the Center for the Diagnosis of Dementia, Kyoto Prefectural University of Medicine, and volunteers who lived locally or were members of an employment service center for older adults. They were aged 60 years or older and had maintained activities of daily living. The participants were recruited from the Center for the Diagnosis of Dementia, including both older adults with or without cognitive impairment, based on various neuropsychological tests. The inclusion criteria for CD were as follows: a score of 0.5 or higher points on the Clinical Dementia Rating (CDR) (18) or 26 or lower points on the Mini-Mental State Examination (MMSE) (15). The inclusion criteria for CND were 0 points on the CDR and 27 or higher points on the MMSE. The exclusion criteria for CD and CND were as follows: a history of mental illness, brain injury, drug or alcohol abuse, and serious impairment in vision, hearing, or the function of both hands. Thus, CD included older adults diagnosed with MCI, mild Alzheimer's disease (AD), or mild vascular dementia. This study was conducted from June 4, 2018, to October 15, 2019, and was approved by the ethics committee of the Kyoto Prefectural University of Medicine (ERB-C-1151-2). Informed consent for participation was obtained from all participants and a legal representative, such as a family member, of one participant.

### Procedures

After finishing the informed consent process, the participants completed the interview, scam vulnerability scale (see Scam Vulnerability Scale), neuropsychological tests (see Neuropsychological Tests), and items related to psychosocial characteristics (see Psychosocial Characteristics) at the University hospital.

### Scam Vulnerability Scale

One consumer counselor, two dementia specialists, one clinical epidemiologist, two clinical psychologists, and one cognitive neuropsychologist extracted 24 questions from 11 aspects in reference to the preliminary interview data from four consultants who were financial scam victims, items from a scam awareness survey, and previous research [e.g., (16)]. We conducted a preliminary survey with seven older adults affected by MCI to examine scale wording. A nine-item scale was finally created, with responses reported using a four-point Likert scale (3 = *Applicable* to 0 = *Not applicable*) for the following statements. The participants answered the items below in Japanese. English translations were guaranteed by the back-translation method.

- I am confident that I will not be scammed.
- If someone I do not know visits, I do not listen to them (reverse item).
- Even if I am dissatisfied with my situation, I am overpowered by my opponent.
- I pick up the phone as soon as I get a call.
- I am interested in tempting offers.
- Even if I think the other person's story is suspicious, I think in a good direction.
- If someone I do not know talks to me in a strong tone, I will be frightened.
- If someone praises or gives special treatment to me, I will be happy.
- I feel anxious about talking to my family and friends about money because it is likely to lead to me losing their trust.

The total score was the average of ratings across the nine items, with the second item reverse coded. Higher scores indicated greater vulnerability to scams for all items.

### Neuropsychological Tests

We used the MMSE to evaluate cognitive function and exclude patients with dementia. The MMSE is a widely used neuropsychological battery for assessing cognitive function and screening for dementia. The total score is 30 points, and the mean score is 27.6 (SD = 1.7) in healthy older adults (19). A low score indicates poor performance.

We used the Japanese version of the Alzheimer's disease assessment scale-cognitive subscale (ADAS-Jcog) to evaluate global cognition (20). A rating of 0 signifies no impairment on a task or the absence of a particular behavior whereas, a rating of 10 is for the most severe or frequent degree of impairment. The maximum score is 70, and a high score indicates poor performance.

We also used the Rivermead Behavioral Memory Test (RBMT) (21, 22) to evaluate functional capacity for independent living and follow up the treatments for everyday memory problems. It is a widely used neuropsychological battery for assessing different types of memory and screening for dementia. The highest score for the RBMT is 24 points, the standard profile score (SPS). The SPS is transformed as a screening score (SS) into dichotomous scores by assigning a value of one to each correct subtest and 0 to each incorrect one. The highest score for SS is 12 points, with a low score indicating poor performance.

The clock drawing test (CDT) is a simple task to administer and is often used to screen for dementia. Cognitive functions, such as visuospatial ability, executive function, comprehension, and semantic memory, are necessary to complete the CDT (23). We presented participants with a blank sheet of paper and a pencil and gave the following instructions: “I would like you to draw a clock that points to 10 after 11” (free-drawn method). We used the Rouleau CDT, commonly used as a CDT scoring system, to score the clock drawings. The Rouleau CDT scores clock drawings for the free-drawn method. The highest score is 10 points, and a low score indicates poor performance.

The verbal fluency test is one of the most widely employed measures to assess cognitive functioning following neurological damage. It involves associative exploration and retrieval of words based on phonemic or semantic criteria. In two separate trials, we asked the participants to generate within 60 sec as many words as possible that begin with the syllable “ka” in the category “vegetables.” We chose this syllable based on a previous study on fluency in Japanese patients with dementia (24). We used the total number of generated unique words as the outcome measure. A low total number of words indicate poor performance.

The Japanese version of the Executive Interview (J-EXIT25) has 25 items and includes tests for assessing frontal lobe function. Each item is scored on a scale of 0–2 points, and the total score ranges from 0 to 50. A higher J-EXIT25 score indicates greater impairment (25, 26). In our study, a high score indicates poor performance.

The Symbol Digit Modalities Test (SDMT) requires participants to pair numbers and symbols verbally according to a fixed pattern. The final score is determined by the number of pairings solved correctly within 90 sec (27). In our study, a low score indicates poor performance.

CDR is derived from a semi-structured interview with the patient and an appropriate informant and rates impairment in six cognitive categories on a five-point scale from 0 = *None* to 3 = *Severe* (18). In our study, a high score indicates poor performance.

### Psychosocial Characteristics

All participants completed the Socio-economic Status Scale (28), living style with family, income satisfaction, frequency of going out, the revised University of California at Los Angeles (UCLA) Loneliness Scale (29, 30), Geriatric Depression Scale-Short Version (31, 32), Multidimensional Scale of Perceived Social Support (MSPSS) (33), Instrumental Activities of Daily Living Scale (IADL) (34), subjective ability for viewing and hearing, and decision making. The decision-making questions included three items. The first was on the emotional framing of medical options. We gave the following instructions: “Which medicine do you think is more dangerous? Medicine A: Five out of 100 people get sick, but the rest get better (negative frame). Medicine B: 95 out of 100 people get better, but the rest get sick (positive frame).” The second was on risk framing in the gain domain. “Which lottery do you choose? Lottery A: You have an 80% chance of getting 4,000 JPY and a 20% chance of getting nothing (uncertain frame). Lottery B: You have a 100% chance of getting 3,000 JPY (certain frame).” The third was on risk framing in the loss domain. “Which lottery do you choose? Lottery C: You have an 80% chance of losing 4,000 JPY and a 20% chance of losing nothing (uncertain frame). Lottery B: You have 100% chance of losing 3,000 JPY (certain frame).”

### Statistical Analyses

To clarify whether missing values were as complete at random, we conducted Little's missing completely at random (MCAR) test. MCAR is a data set in which the missing values occur completely at random. If the missing values are not MCAR, the full information maximum likelihood method (FIML) or the multiple imputation method (MIM) is recommended to deal with the missing values, instead of the list-wise deletion or the pair-wise deletion method. For group differences between CD and CND for each factor, we conducted an unpaired *t*-test for age, education years, income satisfaction, and total scores on the UCLA Loneliness Scale, MSPSS, MMSE, ADAS-Jcog, RBMT-SS (Rivermead Behavioral Memory Test-Screening Score), CDT, verbal fluency, J-EXIT25, and SDMT. We also performed chi-squared tests for sex (female = one, male = two), living style with family, and all decision-making items. We used Mann–Whitney U test for SES, frequency of going out, subjective ability for viewing and hearing, IADL, GDS-S (Geriatric Depression Scale-Short version), and CDR.

To clarify the internal consistency of the scam vulnerability scale, we calculated the Cronbach's α coefficient for nine items and the highest value excluding each item. For the group differences between CD and CND in the scam vulnerability scale, we conducted a Mann–Whitney U test for each item and the total scores of the scam vulnerability scale. Next, we clarified the correlations among scam vulnerability, cognitive function, and psychosocial factors in the CD and CND, respectively, by conducting a hierarchical multiple regression analysis (stepwise method) with the scam vulnerability scale as the dependent variable and the abovementioned twenty variables (age, sex, years of education, living style with family, income satisfaction, frequency of going out, IADL, UCLA, GDS-S, MSPSS, MMSE, SDMT, J-EXIT25, ADAS-Jcog, CDT-free, RBMT-SS, Fluency-letter, and three items of decision making) as the independent variables. For stepwise analysis, the value of the probability of F to enter was 0.05, and to remove was 0.10. Multicollinearity did not occur when independent variables in a regression model were correlated.

## Results

Missing values were found in eight of 101 participants (8%), 19 of 45 variables (35%), and 25 of 5,530 items (0.5%). We conducted MIM for missing values by analyzing them as not MCAR because the results of Little's MCAR test revealed statistically significant differences (χ^2^ (213) = 266.89, *p* < 0.01).

Table 1 shows the group differences between CD and CND for each factor. CD had higher ages (*t* (99) = 3.30, *p* < 0.01, *r* = 0.32) and fewer years of education (*t* (99) = −2.05, *p* < 0.01, *r* = 0.20) compared with CND. CD also recorded lower scores on the MMSE (*t* (59) = −9.23, *p* < 0.001, *r* = 0.77), SDMT (*t* (99) = −3.94, *p* < 0.001, *r* = 0.37), RBMT-SS (*t* (92) = −5.70, *p* < 0.001, *r* = 0.51), and verbal fluency (letter, *t* (99) = −3.49, *p* < 0.01, *r* = 0.33; category, *t* (99) = −3.34, *p* < 0.01, *r* = 0.32), and higher scores in J-EXIT25 (*t* (77) = 4.25, *p* < 0.001, *r* =0.44), ADAS-Jcog (*t* (73) = 5.84, *p* < 0.001, *r* = 0.56), and CDR (U = 346, *p* < 0.001, r = –0.07) compared with CND. Moreover, more CD participants tended to select “I do not know” for emotional negative framing decision making (χ^2^ (3) = 17.93, *p* < 0.001, *V* = 0.42) and risk preference options for gain-domain framing decision making (χ^2^ (1) = 4.49, *p* < 0.05, *V* = 0.21) compared with CND. We found no statistically significant differences in sex (χ^2^(1) = 3.10, *n.s., V* = 0.18), SES (*U* = 1,088, *p* = 0.17, *r* = –0.01), living style with family (χ^2^(4) = 1.34, *n.s., V* = 0.18), income satisfaction (*t* (89) = 0.87, *p* = 0.42, *r* = 0.09), frequency of going out (*U* = 1,206, *p* = 0.54, r = -0.01), subjective ability for viewing (*U* = 1,201, *p* = 0.60, r = –0.01) and hearing (*U* = 1,228, *p* = 0.71, r = –0.01), IADL (*U* = 1,102, *p* = 0.21, r = –0.01), UCLA Loneliness Scale (*t* (99) = 1.03, *p* = 0.30, *r* = 0.10), GDS-S (*U* = 1,131, *p* = 0.33, *r* = –0.01), MSPSS (*t* (99) = 0.70, *p* = 0.49, r = 0.07), CDT-free (*t* (94) = −1.56, *p* = 0.12, *r* = 0.18), and loss-domain framing decision making (χ^2^(1) = 0.88, *p* = 0.35, *V* = 0.09) between the two groups.

**Table 1:** Characteristics of participants in the cognitive decline (CD) and cognitive non-decline (CND) groups.

	**CD**	**CND**	***p* value**
Age	79.4	76.1	0.004^**^
Sex (female)	35	27	0.078^n.s.^
**Psychosocial factors**			
Years of education	12.6	13.6	0.043^*^
SES^a)^	4	4	0.171^n.s.^
Living style with family ^b)^	3	3	0.855^n.s.^
Income satisfaction	3.4	3.3	0.341^n.s.^
Frequency of going out ^a)^	1	1	0.537^n.s.^
Ability for viewing ^a)^	3	3	0.602^n.s.^
Ability for hearing ^a)^	1	1	0.707^n.s.^
IADL^a)^	4	5	0.205^n.s.^
UCLA	30.5	28.7	0.304^n.s.^
GDS-S	3	4	0.327 ^n.s.^
MSPSS	66.2	64.2	0.467^n.s.^
**Neuropsychological factors**			
MMSE	24.0	28.8	<0.001^***^
SDMT	34.1	43.4	<0.001^***^
RBMT-SS	4.9	8.2	<0.001^***^
Fluency-Letter	8.2	11.0	<0.001^***^
-Category	11.0	14.1	0.001^**^
J-EXIT25	11.2	7.3	<0.001^***^
ADAS-Jcog	11.2	4.8	<0.001^***^
CDT-Free	7.3	8.0	0.120^n.s.^
CDR^a)^	0.5	0.0	<0.001^***^
**Decision-making**			
Emotional framing^b)^	4	2	<0.001^***^
Risk framing (gain domain)^b)^	2	2	0.034^**^
Risk framing (loss domain)^b)^	1	1	0.349^n.s.^

*SES, Socio-economic status scale; IADL, Instrumental activities of daily living scale; UCLA, University of California at Los Angeles loneliness scale; GDS-S, Geriatric depression scale-short version; MSPSS, Multidimensional Scale of Perceived Social Support; MMSE, Mini-mental state examination; SDMT, Symbol digit modalities test; J-EXIT25, Japanese versions of the executive interview; ADAS-Jcog, the Japanese version of the Alzheimer's disease assessment scale-cognitive subscale; CDT, Clock drawing test; RBMT-SS, Rivermead behavioral memory test-screening score;*

a) *Median;*

b) *Mode;*

*** *p < 0.001*,

** *p < 0.01*,

* *p < 0.05*,

n.s. *not significant*.

### Reliability of the Scam Vulnerability Scale

Table 2 shows the total mean and mean scores for each item on the scam vulnerability scale. The Cronbach's α coefficient of the nine items of the scam vulnerability characteristics scale was 0.34 (CD = 0.25, CND = 0.40). To create a highly consistent internal scale, we deleted three items (items 1, 2, and 4) and then recalculated the Cronbach's α coefficient, obtaining 0.72 (CD = 0.65, CND = 0.76). To ensure the highest internal consistency in this study, we used six items of the scam vulnerability scale. The total mean and mean scores for each item of the scam vulnerability scale showed no statistically significant differences between the two groups (total for the six-item version, *U* = 1,195, *p* = 0.56, *r* = –0.06; total, *U* = 1,175, *p* = 0.49, *r* = –0.07, item 1, *U* = 1,193, *p* = 0.53, *r* = –0.06; item 2, *U* = 1,271, *p* = 0.98, *r* = –0.01; item 3, *U* = 1,215, *p* = 0.62, *r* = –0.05; item 4, *U* = 1,266, *p* = 0.95, *r* = –0.01; item 5, *U* = 1,158, *p* = 0.35, *r* = –0.09; item 6, *U* = 1,210, *p* = 0.61, *r* = –0.05; item 7, *U* = 1,130, *p* = 0.23, *r* = –0.02; item 8, U = 1,210, *p* = 0.56, r = –0.06; item 9, *U* = 1,149, *p* = 0.29, *r* = –0.00).

**Table 2:** Mean scores of participants in the cognitive decline (CD) and cognitive non-decline (CND) groups in the scam vulnerability scale.

	**CD**	**CND**	***p* value**
Item 1	1.32	1.39	0.53^n.s.^
Item 2	0.88	0.92	0.98^n.s.^
Item 3	0.90	0.86	0.62^n.s.^
Item 4	1.32	1.37	0.95^n.s.^
Item 5	0.66	0.57	0.35^n.s.^
Item 6	0.66	0.61	0.61^n.s.^
Item 7	0.92	0.76	0.23^n.s.^
Item 8	0.88	0.98	0.56^n.s.^
Item 9	0.90	0.84	0.29^n.s.^
Total for the six-item version	4.92	4.62	0.56^n.s.^
Total	8.84	8.61	0.49^n.s.^

n.s. * not significant*.

### Regression on Scam Vulnerability

We conducted regressions on the scam vulnerability scores (Table 3). In CD, the total ADAS-Jcog score (*β* = –0.06, *p* < 0.001) had the greatest effect on vulnerability to scam, and sex (*β* = –0.09, *p* = 0.019) also showed a significant effect. The goodness of fit of the model was R^2^ = 0.32, adjusted R^2^ = 0.29, (*F* (2, 47) = 11.22, *p* < 0.001, *η*^2^ = 0.32) in CD. In CND, sex (*β* = –0.02, *p* = 0.022) had the greatest effect on scam vulnerability. The goodness of fit of the model was R^2^ = 0.10, adjusted R^2^ = 0.08, (*F* (1, 49) = 5.58, *p* = 0.022, *η*^2^ = 0.10) in CND. We also found that inhibition and executive function do not correlate significantly with scam vulnerability.

**Table 3:** Relation of cognitive function and sex to scam vulnerability.

**Variable**	**Model term**	**Estimate (Standard error**, ***p*** **value)**		
		**CD**		**CND**
		**Model 1**	**Model 2**	**Model 1**
Scam vulnerability	ADAS-Jcog	−0.164 (0.045, <0.001 ^***^) (−0.262 ≦ B ≦ −0.086)	−0.164 (0.043, <0.001 ^***^) (−0.248 ≦ B ≦ −0.080)	
	Sex		−1.181 (0.484, 0.015 ^*^) (−2.131 ≦ B ≦ −0.232)	−1.569 (0.664, 0.018 ^*^) (−2.871 ≦ B ≦ −0.268)

*CD, cognitive decline group; CND, cognitive non-decline group; ADAS-Jcog, Japanese version of the Alzheimer's Disease Assessment Scale–Cognitive Subscale, higher score indicates lower cognitive function; Sex, male coded as 0, female coded as 1;*

*** *p < 0.001*,

* *p < 0.05*.

## Discussion

### Scam Vulnerability Scale

This study aimed to determine whether cognitive decline is a risk factor for scam vulnerability in older adults. Thus, we developed a scam vulnerability scale to measure the same. We found no statistical difference in the mean total score of the scam vulnerability scale between CD and CND. Moreover, the scam vulnerability scale with six items obtained sufficient Cronbach's α coefficient in each group, indicating acceptable internal consistency.

### Cognitive Function and Scam Vulnerability

Hierarchical multiple regression analysis revealed that lower ADAS-Jcog scores (higher general cognitive function) correlated with higher scam vulnerability in CD. Moreover, no significant correlation between inhibition or executive function, and scam vulnerability was found. These results suggest that older adults with mild cognitive decline have higher scam vulnerability because older adults with moderate to severe cognitive decline may have difficulty understanding situations and making appropriate decisions. Fraudsters are able to maliciously fill gaps in memory with information that may ultimately benefit them. Mild cognitive decline may provide opportunities for older adults to rely on simpler heuristics. They may also feel pressured to make a rash choice which could increase their vulnerability to frauds and scams (7).

Because the participants in this study included not only older adults with MCI but also those with mild dementia, higher cognitive function correlated with higher scam vulnerability in CD. Han et al. (7) only included patients with MCI and reported a correlation between lower cognitive function and higher scam vulnerability. Although the direction of correlation between cognitive function and fraud vulnerability differed depending on the level of the participants' cognitive function, our findings suggest that mild cognitive decline is a risk factor for scam vulnerability in older adults and that severe cognitive decline is not correlated with scam vulnerability.

The hierarchical multiple regression analysis results revealed that inhibition and executive function do not correlate with higher scam vulnerability. Wood et al. (9) reported that older adults who were victims of financial exploitation show poorer executive functioning than the control group. Their study focused on financial exploitation, not specific frauds and scams. Inhibition and executive function can be correlated depending on the scam content such as using rewards or time limits in lottery or phishing schemes. Thus, there is a gap in the research on the correlation of inhibition and executive function with frauds and scams victimization.

### Psychosocial Characteristics and Scam Vulnerability

We found a higher correlation between the male sex and scam vulnerability in both CD and CND. According to the National Police Agency in Japan, women account for 65.3% of all frauds and scams victims aged over 65 years old (1). Per a national survey of telemarketing fraud by the American Association of Retired Persons, the typical lottery victim is a woman over 75 years old, widowed and living alone, retired, with a household income lower than 30,000 USD (35). Meanwhile, Alves and Wilson (13) reported that telemarketing fraud victims tend to be male and between the ages of 60 and 70. However, Lichtenberg et al. (11, 12), Judges et al. (8), and James et al. (16) found no correlation between fraud victimization or vulnerability and sex differences. Thus, the evidence on sex-related differences in fraud and scam victimization or vulnerability remains inconsistent. Men may report higher vulnerability and lower victimization and women, lower vulnerability and higher victimization because socially disadvantaged consumers often do not report their victimization (36). That is, men who are more vulnerable to fraud are less likely to report actual victimization experiences. Another possibility is that because older women in Japan are more likely to be housewives staying at home during the day and responsible for managing their household budget, they tend to answer phone calls at home or door-to-door sales visits, thereby falling prey to scammers more easily.

Further, our hierarchical multiple regression analysis results showed no psychosocial characteristics that are correlated with higher scam vulnerability in either group. According to previous research, frauds and scams victimization or vulnerability are correlated with being younger, having a higher level of education, having a higher level of depression, having more social activities, taking higher risks, having lower honesty–humility and conscientiousness, being overconfident in financial knowledge, having lower well-being, and possessing less economic and health literacy (8, 11–16). Our study examined not only psychosocial characteristics but also cognitive function in older adults with or without cognitive decline, and we found that cognitive decline is a strong risk factor for scam vulnerability in older adults, including those with MCI, mild AD, and vascular dementia.

### Limitations

The present study has some limitations. First, the scam vulnerability scale was developed based on preliminary interview data from financial scam victims, items from a scam awareness survey, and previous research. In the present study, healthy older adults and older adults with cognitive decline could use this scam vulnerability scale to assess their traits for scam vulnerability. However, future studies need to examine the scale validity in cross-sectional populations between fraud victims and non-victims or longitudinal data on fraud experiences. Second, we examined the factors that are correlated with vulnerability to scam; thus, the present results predicted scam vulnerability, not victimization. The differences in predictive factors between frauds and scams vulnerability and victimization merit further research. Moreover, it is necessary to evaluate the cognitive function in victims of frauds and scams and examine whether cognitive decline is associated with fraud and scam victimization. Third, our study did not examine all risk-related factors of psychosocial characteristics, such as the personality traits of lower honesty–humility and conscientiousness, and financial and health literacy. Future studies should investigate other risk-related factors in older adults with cognitive impairment.

### Conclusion

The current results provide new insights into the relationship between cognitive decline and fraud vulnerability in older adults. The total ADAS-Jcog score, as a global cognitive function, had the most effect on scam vulnerability in CD. The male sex also significantly affected scam vulnerability in both CD and CND groups. Meanwhile, we did not find a significant correlation between scam vulnerability and the participants' inhibition and executive function. Thus, mild cognitive decline in global cognitive function is a risk factor for scam vulnerability in older adults, but severe cognitive decline shows no correlation. Moreover, older adults with mild cognitive decline and their families, particularly those visiting elderly care or outpatient facilities, should be notified of the scam vulnerability of older clients using the ADAS-Jcog score as an index to help them avoid victimization.

## Data Availability Statement

The raw data supporting the conclusions of this article will be made available by the authors, without undue reservation.

## Ethics Statement

The studies involving human participants were reviewed and approved by Kyoto Prefectural University of Medicine (ERB-C-1151-3). The patients/participants provided their written informed consent to participate in this study.

## Author Contributions

DU: execution of the research project, data collection, statistical analysis, writing the first draft, and manuscript review and critique. YD: execution of the research project, data management, statistical review and critique, and manuscript review and critique. YE and MI: execution of the research project, statistical review and critique, and manuscript review and critique. SA: assistance in the execution of the research project, data collection, and manuscript review and critique. NA and TM: statistical review and critique and manuscript review and critique. KN: data collection and manuscript review and critique. YK: manuscript review and critique. JN: organization and execution of the research project, data collection, statistical review and critique, and manuscript review and critique. All authors read and approved the final manuscript.

## Funding

This study was based on work supported by JST, RISTEX Grant Number JPMJRX17G1, Japan.

## Conflict of Interest

The authors declare that the research was conducted in the absence of any commercial or financial relationships that could be construed as a potential conflict of interest.

## Publisher's Note

All claims expressed in this article are solely those of the authors and do not necessarily represent those of their affiliated organizations, or those of the publisher, the editors and the reviewers. Any product that may be evaluated in this article, or claim that may be made by its manufacturer, is not guaranteed or endorsed by the publisher.
